# Differences in Pathologic Results of Repeat Transurethral Resection of Bladder Tumor (TURBT) according to Institution Performing the Initial TURBT: Comparative Analyses between Referred and Nonreferred Group

**DOI:** 10.1155/2018/9432606

**Published:** 2018-09-06

**Authors:** Hyeong Dong Yuk, Jung Kwon Kim, Chang Wook Jeong, Cheol Kwak, Hyeon Hoe Kim, Ja Hyeon Ku

**Affiliations:** ^1^Department of Urology, Seoul National University Hospital, Seoul, Republic of Korea; ^2^Department of Urology, Seoul National University Bundang Hospital, Seongnam, Republic of Korea

## Abstract

**Objective:**

Although transurethral resection of bladder tumor (TURBT) is a standard treatment and determines staging for nonmuscle invasive bladder cancer, many deficiencies persist. There is a risk of upstaging and residual cancer when repeat TURBT is performed. Authors compared the results of repeat TURBT by institution performing the initial TURBT.

**Methods:**

We retrospectively reviewed the medical records of 289 patients who underwent repeat TURBT within 2-6 weeks after initial TURBT between 1998 and 2013. The patients were divided into the referred group and the nonreferred group by institution performing the initial TURBT. And we analyzed the intergroup differences in residual tumor and upstaging rate and the factors significantly correlated with residual tumor.

**Results:**

The mean age was 69.6 ± 11.1 years and the mean follow-up was 49.7 (range: 0–191) months. The referred group included 69 patients, while the nonreferred group included 220 patients. The referred group included 57 (82.6%) patients with residual tumor after repeat TURBT. Overall upstaging occurred in 15 (21.7%), and upstaging to T2 occurred in 11 (15.9%) of the initial Ta and T1 patients. In the nonreferred group, there were 123 (55.9%) patients with residual tumor. Overall upstaging occurred in 10 (4.5%) and upstaging to T2 occurred in 7 (3.2%) patients.

**Conclusions:**

Gross hematuria, grade, and tumor quantity and size were significantly associated with residual cancer on multivariate analysis. In the referred group, repeat TURBT and restaging are necessary.

## 1. Introduction

Bladder cancer, the seventh most common cancer in the world [1], is highly prevalent in the United States, Europe, and Egypt. More than 400,000 people are diagnosed every year worldwide [2]. Approximately 75–85% of bladder cancer patients have nonmuscle invasive bladder cancer, for which transurethral resection of bladder tumor (TURBT) is the standard treatment.[3] As with any cancer, staging accuracy is important because treatment can vary depending on pathology results. Stage is determined by histology, grade, and invasion depth. Depending on stage, treatment methods such as TURBT, intravesical Bacillus Calmette–Guérin vaccine, chemoagent instillation, and radical cystectomy are used.[4] Staging can be determined through TURBT; however, the accuracy is not always precise since tumors might not be immediately visible under the mucosa.[5] In cases of such invisible tumors, exact extent and depth cannot be precisely determined. Therefore, there is a risk of upstaging and residual cancer when repeat TURBT is performed. Many previous studies have discussed the importance of repeat TURBT for this reason. In cases of incomplete initial TURBT, no muscle in the specimen after the initial resection (with the exception of TaG1 tumors and primary carcinoma in situ [CIS]), all T1 tumors, and all HG/G3 tumors (except primary CIS), The European Association of Urology (EAU) guideline recommends repeat TURBT. [4] Most previous studies were the results of repeat TURBT after initial TURB in the same hospital. [6–10] There are few reports of repeat TURBT result for patients after initial TURBT in another hospital. [11, 12] Therefore, we would like to compare the outcomes of cases of initial and repeat TURBT in a single hospital to those of repeat TURBT in one hospital after initial TURBT in a different hospital.

## 2. Materials and Methods

### 2.1. Study Sample

The Institutional Review Board at our center approved this study (H1704-149-848). We conducted a retrospective study. It was exempt from the requirement of informed consent from the patient. All research plans and related research plans followed the Helsinki Declaration Guidelines. We analyzed the medical records of 289 patients who underwent repeat TURBT at Seoul National University Hospital (SNUH) between 1998 and 2013. The selection criteria are as follows. Cases of initial and repeat TURBT intervals 2-6 weeks. The exclusion criteria are as follows. We excluded patients with incomplete early TURBT with massive mass, patients with distant metastasis before surgery, patients with upper urinary tract cancer, and patients who underwent preoperative chemotherapy.

### 2.2. Study Design

The patients were divided into the referred group and the no-referred group by institution performing the initial TURBT. The nonreferred group performed both initial TURB and repeat TURB in SNUH. In the referred group, initial TURB was performed at another hospital and repeat TURB was performed at SNUH. Most of referred group patients came to SNUH for a second opinion after undergoing an initial TURBT in another hospital. Some of the referred group patients were referred for radical cystectomy at other hospitals. All patients provided their previous medical records and pathological slides obtained with the initial TURBT. Patients in the nonreferred group underwent repeat TURBT according to EAU guidelines after the initial TURBT here at SNUH. Repeat TURBT was performed with removal of the previous TURBT site including the muscularis propria layer and resection of the suspected site. In addition, cold cup biopsies were performed including six sites of anterior wall, posterior wall, both lateral walls, dome, trigone, and prostate. All patients underwent general or spinal anesthesia. Postoperative records included tumor location, size, shape, and quantity. Pathologic classification was performed by experienced two genitourinary pathologists.

### 2.3. Statistical Analysis

We compared tumor remaining and upstaging rates between the referred and nonreferred groups. The primary end point is the residual tumor and the secondary end point is the upstaging. The continuous variables were expressed as mean values with standard deviation (SD) or quartile range (IQR), and categorical variables were presented as percentage of incidents. Each group was analyzed for residual tumor rate, overall upstaging rate, and conversion rate to T2 according to initial TURBT stage (Ta and T1). We analyzed factors related to presence of residual tumor using univariate and multivariate logistic regression analysis. Statistical significance was considered when the p value was less than 0.05. IBM SPSS Statistics version 22.0 (IBM, Armonk, New York, USA) was used.

## 3. Results and Discussion

The total number of patients was 289: 69 in the referred group and 220 in the nonreferred group. The referred group consisted of patients referred from 32 regional hospitals and 26 university hospitals. 31 patients from regional hospitals and 38 patients from university hospitals visited for a voluntary second opinion and treatment. The mean age was 69.6 ± 11.1 years and the mean follow-up period was 49.7 (range: 0–191) months. The results of the initial TURBT pathology were as follows. The nonreferred group had 33 (15%) and 187 (85.0%) patients with Ta and T1, respectively, while the referred group had 15 (21.7%) and 54 (78.3%) patients with Ta and T1, respectively. Patients with CIS comprised 20 (9.0%) in the nonreferred group and 4 (5.8%) in the referred group. There were 113 (51.3%) and 35 (50.7%) patients with multifocal tumors, and 68 (30.9%) and 14 (20.3%) patients had tumors > 3 cm (Table 1). Residual tumor was reported in 123 (55.9%) of 220 patients who underwent repeat TURBT in the nonreferred group, overall upstaging was reported in 10 patients (4.5%), and upstaging to T2 occurred in 1 (3.0%) and 6 (3.2%) patients with initial Ta and T1. Of the 69 patients in the referred group, 52 (82.6%) reported residual cancer and overall upstaging was reported in 15 (21.7%). Upstaging to T2 occurred in only 11 (15.9%) patients with T1 (Tables 2 and 3).

We used both univariate and multivariate logistic regression models to analyze the factors associated with residual tumors after initial TURB. We analyzed sex, body mass index, muscularis propria layer inclusion, period of primary recurrence, number of previous TURBT procedures, previous intravesical therapy, and T stage as factors related to residual tumor. Gross hematuria, 2004 WHO/ISUP grade (low vs. high) (p = 0.006), concomitant CIS (p = 0.011), and tumor quantity (p = 0.014) were significantly associated with residual tumor after repeat TURBT on univariate analysis. The gross hematuria included in the analysis is the presence of initial symptoms before initial TURB. When univariate and multivariate analysis were performed, gross hematuria, number of previous TURBT, tumor grade, tumor multiplicity, concomitant CIS, and tumor size referred status were significant factors to affect residual tumors (Table 4). And in multivariate analysis of factors affecting upstaging, T stage, number of previous TURBT, and LVI were important factors (Table 5).

Although TURBT is a standard treatment for nonmuscle invasive bladder cancer, many deficiencies persist. Many studies have reported that clear resection does not work well, residual tumor remains, and understaging is common [6, 7, 11]. Therefore, restaging reportedly requires repeat TURBT [8, 13]. Various studies reported residual tumor after repeat TURBT in 27.3–77.6% and upstaging in 1.7–33.3% of cases [8–15].

Here we reported on 289 patients who underwent repeat TURBT after initial TURBT. Patients with previous recurrent history of TURBT were 17 (7.7%) in the nonreferred group and 60 (87.0%) in the referred group. There were many patients with recurrent repeat TURBT records in the referred group, and the ratios of T1 to T2 were 6 (3.2%) and 11 (20.4%), respectively. These results suggest that the referred group had more relapses after TURBT and more T2 patients than the nonreferred group. Because of dissatisfaction with previous treatment or the recommendation of radical cystectomy for T2, the patients often visited a larger hospital for a second opinion or other treatment. The rates of residual tumor were 57 (82.6%) in the referred group and 123 (55.9%) in the nonreferred group when repeat TURBT was performed. The rates of residual tumor after repeat TURBT were 27.3–72.3% for Ta, 32.9%–77.6% for T1, 6.5–33.3% for Ta upstaging, and 1.7–23.3% for T1 in various studies [10–12, 15].

Overall, residual tumor, overall upstaging, and upstaging to T2 were higher in the referred group than in the nonreferred group. No previous study compares referred and nonreferred groups. Han and Herr studied referred patients and showed a 64–77.6% residual tumor rate and an 8–33.3% upstaging rate [11, 12]. However, in the Schips and Zukirchen studies of nonreferred patients, the residual tumor rate was 27.3-38.7% and the upstaging rate was 1.7–18.5% [10, 15]. Although the comparison is difficult due to the lack of the same conditions, these studies show that the numerical values tend to be higher in the group as in our study (Table 6) [10–12, 15]. The intergroup differences are considered to reflect the difference in surgeon proficiency and experience of the initial TURBT stage. Specimen quality can vary by surgeon experience as well as tumor size, multiplicity, and lesion. Differences in the quality of these specimens affect diagnosis [16, 17]. And the mean stage is likely to be higher than that of the nonreferred group.

The high rate of residual tumor and upstaging in the referred group compared to the nonreferred group indicates that repeat TURBT and restaging are usually needed for referred patients. Some studies recommend active surveillance for Ta stage or reconsideration of the need for repeat TURBT [18–20]. However, the upstaging rate in patients with initial Ta was 8–33% in various studies [10–12, 15]. In our study, the upstaging rate was 16.6% overall, and in some cases upstaging for T2 and repeat TURBT for Ta stage is considered necessary.

In the present study, factors associated with residual tumor after repeat TURB were gross hematuria, grade, concomitant CIS, and tumor quantity on univariate analysis and gross hematuria, grade, tumor quantity, and tumor size on multivariate analysis. Cano-Garcia and Donat also indicated a significant relationship between residual tumor and size [21, 22]. Kamiya et al. suggested that tumor multiplicity may be a related factor in residual tumors [23]. Considering repeat TURBT, these factors may be helpful. Several tumor markers have recently been approved by the FDA for early diagnosis of bladder cancer [24]. These tumor markers will have higher sensitivity than urine cytology and will replace the cystoscopy test. Cystoscopy is less effective and invasive than urinary biomarker test. The development of this biomarker will be associated with high grade NMIBC diagnosis and contribute to the differentiation of residual tumor and accuracy of staging [25–27].

There are several limitations of this study. First, this is a single center observational study. Second, there may be selection bias due to the retrospective nature. Third, there may be sampling bias due to variability among the institutions and relatively long data collection period.

## 4. Conclusions

This study is the first comparison between the referred and the nonreferred groups with repeat TURBT. The referred group has a relatively higher initial TURBT stage, residual tumor, overall upstaging, and upstaging to T2 than the nonreferred group. It is thought that this is due to the difference of the operator's experience and the initial stage of the two groups. In the referred group, repeat TURBT and restaging are necessary, while repeat TURBT for Ta stage cancer is also necessary. The factors associated with residual tumor after initial TURB were gross hematuria, grade, concomitant CIS, tumor quantity, and tumor size. These factors may be helpful in determining repeat TURBT.

## Figures and Tables

**Table 1:** Characteristics of patients at the initial transurethral resection.

Clinical characteristics	Non-referred group	Referred group	P value
Number of patients	220	69	

Sex			0.998

Male	186 (84.5%)	59 (85.5%)	

Female	34 (15.5%)	10 (14.5%)	

BMI	23.9±2.9	24.0±3.0	0.878

Gross hematuria	173 (79.0%)	53 (76.8%)	0.828

Number of previous TURBT			<0.001

0	203 (92.3%)	9 (13.0%)	

1	9 (4.1%)	52 (75.4%)	

≥2	8 (3.6%)	8 (11.6%)	

Previous intravesical therapy	6 (2.7%)	9 (13.0%)	0.002

T stage			0.260

Ta	33 (15.0%)	15 (21.7%)	

T1	187 (85.0%)	54 (78.3%)	

Tumor grade			0.779

Low	32 (14.5%)	21 (30.4%)	

High	188 (85.5%)	48 (69.6%)	

Tumor multiplicity			<0.001

1	107 (48.7%)	34 (49.3%)	

2-7	89 (40.4%)	30 (43.5%)	

>8	24 (10.9%)	5 (7.2%)	

Tumor size			<0.001

< 3cm	152 (69.1%)	55 (79.7%)	

≥ 3cm	68 (30.9%)	14 (20.3%)	

Concomitant CIS	20 (9.0%)	4 (5.8%)	0.006

LVI	9 (4.1%)	0 (0.0%)	0.190

BMI: body mass index; TURBT: transurethral resection of bladder tumor; CIS: carcinoma in situ; LVI: lymphovascular invasion.

**Table 2:** Residual tumor and upstaging results after repeat TURBT in patients.

	Non referred group (N=220)	Referred group (N=69)	P value
Ta at initial TURBT			

Number of patients	33	15	

Residual tumor	19(57.6%)	10 (66.7%)	0.210

Overall upstaging	4 (12.1%)	4 (26.7%)	0.551

Upstaging to T2	1 (3.0%)	0 (0%)	0.496

No residual tumor	14 (42.4%)	5 (33.3%)	0.210

T1 at initial TURBT			

No. of patients	187	54	

Residual tumor	104 (55.6%)	47 (87.0%)	<0.001

Overall upstaging	6 (3.2%)	11 (20.4%)	<0.001

Upstaging to T2	6 (3.2%)	11 (20.4%)	<0.001

No residual tumor	83 (44.4%)	7 (13.0%)	<0.001

Overall			

No. of patients	220	69	

Residual tumor	123 (55.9%)	57 (82.6%)	<0.001

Overall upstaging	10(4.5%)	15 (21.7%)	<0.001

Upstaging to T2	7 (3.2%)	11 (15.9%)	<0.001

No residual tumor	97 (44.1%)	12 (17.3%)	<0.001

TURBT: transurethral resection of bladder tumor.

**Table 3:** Pathologic results after repeat TURBT in patients.

Non-referred group		T stage after re-TURBT (%)				

Initial T	N	T0	Ta	T1	T2	CIS

Ta	33	14 (42.4%)	11 (33.3%)	3 (9.1%)	1 (3.0%)	4 (12.1%)

T1	187	83 (44.4%)	40 (21.4%)	41 (21.9%)	6 (3.2%)	17 (9.1%)

Referred group						

Initial T	N	T0	Ta	T1	T2	CIS

Ta	15	5 (33.3%)	4 (26.7%)	4 (26.7%)	0 (0%)	2 (13.3%)

T1	54	7 (13.0%)	4 (7.4%)	24 (44.4%)	11 (20.4%)	8 (14.8%)

TURBT: transurethral resection of bladder tumor.

**Table 4:** Factors of residual tumor at the repeat TURBT according to univariate and multivariate logistic regression models.

	Univariate			Multivariate		
	OR	95% CI	p-value	OR	95% CI	P value
Sex	1.36	0.70-2.76	0.381	1.05	0.47-2.39	0.913

BMI	0.95	0.88-1.03	0.233	0.98	0.89-1.07	0.625

Number of previous TURBT			<0.001			0.016

0	Reference			Reference		

1	10.38	4.00-26.95	<0.001	6.49	1.35-31.06	0.019

2	6.49	1.44-29.26	0.015	11.56	1.45-92.10	0..021

Previous intravesical therapy	1.22	0.42-4.02	0.719	0.10	0.01-0.65	0.017

Gross hematuria	1.90	1.07-3.36	0.027	2.457	1.245-4.850	0.010

T stage (Ta/T1)	0.91	0.49-1.74	0.770	1.06	0.45-2.51	0.901

Tumor grade at initial TURBT (Low/High)	3.05	1.56-6.13	<0.001	2.56	1.13-6.04	0.026

Concomitant CIS (yes/no)	8.23	2.86-34.80	<0.001	8.06	2.43-37.62	0.002

LVI	2.16	0.51-14.71	0.341	2.51	0.50-18.91	0.298

Tumor multiplicity at initial TURBT						0.029

1	Reference			Reference		

2-7	1.86	1.07-3.24	0.027	2.06	1.11-3.81	0.021

≥8	3.21	1.26-8.14	0.014	2.85	1.05-7.73	0.040

Tumor size at initial TURBT (<3 cm/≥3 cm)	1.88	1.13-3.18	0.016	1.925	1.014-3.656	0.045

Referred/Non-referred status	3.75	1.96-7.68	<0.001	3.47	1.03-11.20	0.036

BMI: body mass index; TURBT: transurethral resection of bladder tumor; CIS: carcinoma in situ; LVI: lymphovascular invasion.

**Table 5:** Factors of upstaging at the repeat TURBT according to univariate and multivariate logistic regression models.

	Univariate			Multivariate		
	OR	95% CI	P value	OR	95% CI	p-value
Sex	0.35	0.08-1.03	0.094	0.27	0.06-0.85	0.045

BMI	1.00	0.0-1.12	0.961	1.02	0.90-1.15	0.793

Number of previous TURBT			0.005			0.048

0	Reference			Reference		

1	1.75	0.82-3.72	0.144	1.15	0.29-4.64	0.838

2	5.56	1.91-16.21	0.002	5.36	1.21-23.78	0.027

Previous intravesical therapy	2.03	1.37-3.05	<0.001	0.76	0.15-3.18	0.718

Gross hematuria	2.45	1.00-7.36	0.071	3.18	1.20-10.27	0.031

T stage (Ta/T1)	2.10	0.96-4.37	0.050	3.32	1.30-3.47	0.011

Tumor grade at initial TURBT (Low/High)	2.58	0.88-11.03	0.127	3.74	1.06-18.40	0.063

Concomitant CIS (yes/no)	1.93	0.80-4.30	0.120	1.24	0.46-3.11	0.656

LVI	1.52	0.97-2.37	0.065	1.38	0.82-2.32	0.218

Tumor multiplicity at initial TURBT			0.231			0.391

1	Reference			Reference		

2-7	1.60	0.80-3.19	0.186	1.63	0.76-3.47	0.209

≥8	2.13	0.80-5.68	0.132	1.77	0.57-5.53	0.326

Tumor size at initial TURBT (<3 cm/≥3 cm)	1.14	0.59-2.16	0.696	1.48	0.66-3.26	0.339

Referred/Non-referred status	1.76	0.87-3.47	0.108	0.86	0.35-2.03	0.732

BMI: body mass index; TURBT: transurethral resection of bladder tumor; CIS: carcinoma in situ; LVI: lymphovascular invasion.

**Table 6:** Pathologic results after repeat TURBT about residual tumor and upstaging.

	Number of patients	Residual tumor (%)		Upstaging (%)	
Initial TURBT at another hospital		Ta	T1	From Ta	From T1

Herr [11]	76	72.2%	77.6%	33.3%	27.6%

Han [8]	55	64.0%	66.7%	8.0%	23.3%

Referred group	104	64.5%	80.4%	26.7%	20.4%

Initial TURBT at same hospital		Ta	T1	From Ta	From T1

Schips [10]	107	38.7%	32.9%	6.5%	7.9%

Zukirchen [15]	214	27.3%	36.5%	18.5%	1.7%

Non referred group	237	26.7%	20.4%	12.1%	3.2%

TURBT: transurethral resection of bladder tumor.

## Data Availability

Data used to support the findings of this study are restricted by the Seoul National University Hospital Clinical Research Institute in order to protect Patient Personal Information. Data are available from Seoul National University Hospital Clinical Research Institute for researchers who meet the criteria for access to confidential data.
